# Multiple insights call for revision of modern thermodynamic models to account for structural fluctuations in water

**DOI:** 10.1002/aic.17891

**Published:** 2022-09-05

**Authors:** Evangelos Tsochantaris, Aswin V. Muthachikavil, Baoliang Peng, Xiaodong Liang, Georgios M. Kontogeorgis

**Affiliations:** ^1^ Department of Chemical and Biochemical Engineering, Center for Energy Resources Engineering Technical University of Denmark Lyngby Denmark; ^2^ Research Institute of Petroleum Exploration & Development (RIPED), PetroChina Beijing China

**Keywords:** thermodynamic models, molecular simulations, SAFT, structural fluctuations, water

## Abstract

Modern thermodynamic models incorporate the concept of association (hydrogen bonding) and they can describe very satisfactorily many properties of water containing mixtures. They have not been successful in representing water's anomalous properties and this work provides a possible explanation. We have analyzed and interpreted recent experimental data, molecular simulation results, and two‐state theory approaches and compared against the predictions from thermodynamic models. We show that the dominance of the tetrahedral structure implemented in modern thermodynamic models may be the reason for their failure for describing water systems. While this study does not prove the two‐state theories for water, it indicates that a high level of tetrahedral structure of water is not in agreement with water's anomalous properties when used in thermodynamic models.

## 1 INTRODUCTION

During the 20th century, thermodynamics has undergone various stages, since the development of simple equations of state and theories. The underlying assumption in these models is that physical forces are the main cause of non‐ideality ignoring association effects (hydrogen bonding). Since the 1980s a new family of advanced equations of state has appeared in literature, using diverse theoretical frameworks. Common with all these models is that both physical and association/hydrogen bonding are explicitly accounted for. Both classical and advanced models are reviewed in textbooks.^1^, ^2^ These models have been highly successful in describing phase equilibria and other properties for hydrogen bonding mixtures. They are not able to represent the anomalous properties of water such as maximum of density, minimum of heat capacity,^3^ although there are some recent promising efforts.^4^ These thermodynamic models assume that water is a homogeneous liquid, largely having a tetrahedral structure, which depends on temperature. Some spectroscopic data from literature available for water's structure^5^ have been contested and most of these models were not in good agreement with these data, indicating that water is much more “hydrogen bonding” (smaller monomer fractions) compared with these spectroscopic data.^6^, ^7^

Recently, there have been significant new developments in water science with the advent of the two‐state theory in new forms^8^, ^9^, ^10^, ^11^, ^12^, ^13^, ^14^, ^15^, ^16^ as well as detailed molecular simulation studies, some of which indicating the existence of a second critical point with various force fields for water.^17^, ^18^, ^19^ This theory suggests the existence of a low density liquid (LDL) water state and a high density liquid (HDL) water state in liquid water. It is important to note that the terms LDL and HDL usually correspond to macroscopic phases which exist only below the theorized liquid–liquid critical point (LLCP). Above the LLCP, there are no two macroscopic phases, but there are structural fluctuations between high‐density local structures and low‐density local structures. Some authors refer to these low density structures as locally‐favored tetrahedral structures (LFTS),^20^ while some other authors simply use the terms LDL and HDL for these two structures, and the macroscopic phases (as applicable).^12^, ^21^ In this study, we refer to the low‐density and high‐density local structures using the terms LDL and HDL, respectively. The reader should note that, when we use these terms, we are not referring to the macroscopic phases of water, but to the local structures. The LFTS fraction (also sometimes referred as LDL fraction by some authors^12^, ^21^) is considered as the tetrahedral fraction of water. No specific structure is typically assigned to HDL structure, sometimes referred to as unstructured water fraction.

There have been many excellent works that supported the idea of liquid–liquid phase transition in water at the molecular level. Using molecular simulations of the ST2 water model, Palmer et al. provided evidence for a liquid–liquid phase transition in liquid water.^22^ They showed the free energy landscapes of liquid–liquid phase transition between two liquid phases and a stable crystal phase of water, further supporting the proposal of two liquid phases in water. There have been arguments that the ST2 water model over‐estimates the ordering in water. They, therefore, used two more realistic water models (TIP4P/Ice and TIP4P/2005) to show the presence of a LLCP.^18^ Recently, Martelli investigated the contribution of the local structures in liquid water towards the anomalies in water,^23^ using molecular simulations of the TIP4P/2005 water model. They reported the cluster sizes, compositions, and hydrogen bonded network of LDL‐like environments. Their results also indicated that water's anomalies may be attributed to the clusters of the LDL‐like and HDL‐like environments and their spatial organization in liquid water. Stokely et al.^24^ have investigated four different scenarios for the behavior of supercooled water (the stability limit, the LLCP, the singularity‐free and the critical point free scenario) using mean‐field calculations and Monte‐Carlo simulations. The models heavily relied on hydrogen bonding interactions and cooperativity effects. They showed that the models are able to represent all the different scenarios. Depending on the strength of the hydrogen bond and of the cooperativity effects, they would produce results that follow different scenarios. Thermodynamic anomalies (like the density maximum) were not shown in that study. Martelli et al.^25^ investigated the connection between liquid and non‐crystalline phases in water as well as their connection to some of water's thermodynamic anomalies. For their study they used molecular simulations of water with the TIP4P/2005 interaction potential and they developed a Neural Network scheme which is capable of discerning different local structures. With this method they found structures in liquid TIP4P/2005 water that resemble the structures of high density amorphous ice and low density amorphous ice. When their population changes dramatically so do the thermodynamic properties of water leading to extrema.

As discussed, there are plenty of studies available in the literature that have investigated the structural fluctuations in water, and their connection to water's thermodynamic anomalies and/or phase‐behavior in the super‐cooled regime. The purpose of our work is to contribute to these discussions, by taking into account different approaches/models (including molecular simulations, experiments and thermodynamic models). For the sake of this discussion, we considered models that include structural fluctuations as well as models that assume a homogeneous structure for liquid water. We compare these models with experimental data and molecular simulation results, for thermodynamic properties and structural properties. From these comparisons, we highlight the importance of the structural fluctuations for the thermodynamic anomalies. Moreover, indirectly, this work contributes to the discussion about the validity of the two‐state theory for water, a theory discussed extensively during the 21st century^26^ and even during the 20th century,^27^, ^28^ but, despite much evidence, still contested and debated.^26^, ^29^, ^30^, ^31^, ^32^, ^33^, ^34^

## 2 MATERIALS AND METHODS

### 2.1 Thermodynamic models

#### 2.1.1 Perturbed chain‐statistical associating fluid theory

Thermodynamic models are useful since they can be used for the prediction and description of thermodynamic properties and phase equilibria. Some noteworthy thermodynamic models are equations of state. Industry uses cubic equations of state because of their simplicity and relative accuracy.^35^, ^36^ However, they are inadequate for substances containing strongly associating molecules, such as water, since they do not consider hydrogen‐bonding.^36^ For these substances, advanced thermodynamic models that take into account hydrogen‐bonding, such as the statistical associating fluid theory (SAFT), would be more viable choices. Chapman et al.^37^, ^38^ derived the SAFT equation of state from Wertheim's thermodynamic perturbation theory.^39^, ^40^, ^41^, ^42^ The SAFT equation of state has a more realistic representation of intermolecular forces compared with the classical cubic ones and there are structural details in the model mainly in the form of the pair‐distribution function of the system and the clusters of associated molecules. Over the past years, many modifications have been suggested for the SAFT model.^43^ The Perturbed Chain‐Statistical Associating Fluid Theory (PC‐SAFT) equation of state is a modified version of the original SAFT model and it was developed by Gross and Sadowski^43^ by deriving a new dispersion term based on the perturbation theory of Barker and Henderson.^44^, ^45^ The dispersion term in PC‐SAFT refers to dispersion forces between whole chains in contrast to the dispersion term of the original SAFT equation, which accounts for dispersion forces between hard spheres. The reduced residual Helmholtz free energy for mixtures containing associating fluids in PC‐SAFT can be expressed from the following equation^46^:

(1) $$a^{r}=a^{\mathrm{hs}}+a^{\text{chain}}+a^{\text{disp}}+a^{\text{assoc}}$$

where *a* ^hs^ is the Helmholtz free energy contribution of the hard sphere segment–segment interaction, *a* ^chain^ is the contribution from covalent chain‐forming bonds, *a* ^disp^ is the contribution from the dispersive forces between whole chains, and *a* ^assoc^ is the contribution from association forces between segments. All the energy contributions are dimensionless.

The various terms of PC‐SAFT are well documented in various articles.^43^, ^46^, ^47^ For this reason, most of these terms will not be shown here. The dispersion forces are expressed using a modified square well pair potential potential^43^ and the hydrogen bonds using a conical square‐well potential (the sites form hydrogen bonds when they have the proper orientation and the energy of H‐bond is constant).^38^ The association term in SAFT‐type models can be expressed as:

(2) $$a^{\text{assoc}}=\sum_{i}x_{i}\left(\sum_{A_{i}}\left(\ln X_{A_{i}}-X_{A_{i}}/2\right)+\frac{1}{2}M_{i}\right)$$

where $X_{A_{i}}$ is the fraction of the molecules *i* not bonded at site *A* and *M*_*i*_ is the number of association sites on molecule *i*. For the calculation of $X_{A_{i}}$, one will need to consider the probability that two association sites will have the correct orientation and the energy of the H‐bond. More details can be found in the original publications.^37^, ^38^

In this study, the simplified PC‐SAFT proposed by von Solms et al.^48^ is used. In their work, they propose two main modifications that simplify the expression of the hard sphere term *a* ^hs^ and the expression of the radial distribution function of the hard sphere reference fluid *g* ^hs^. It is worth noting that for pure compounds the results of original PC‐SAFT and of the simplified PC‐SAFT are identical.

For the application of this equation of state five pure‐component parameters need to be specified for each associating fluid (or three for non‐associating ones). These parameters are typically fitted simultaneously to vapor pressure and saturated liquid density data. More extensive information on the PC‐SAFT equation of state are available in the original literature,^43^ and more information on the modifications are available in the work of von Solms et al.^48^ Parameter sets for PC‐SAFT used in this work along with a short description of each parameter can be found in Table S1 of Supplementary Material.

#### 2.1.2 Cubic‐plus‐association

The cubic‐plus‐association (CPA) equation of state, developed by Kontogeorgis et al.,^49^ combines the classical Soave–Redlich–Kwong (SRK) equation with an association term similar to the association term of the SAFT EOS. For non‐associating fluids, the CPA EOS is reduced to SRK. The CPA can be expressed in terms of residual Helmholtz free energy as^50^:

(3) $$a^{r}=a^{\mathrm{SRK}}+a^{\text{assoc}}$$

where *a* ^SRK^ is the Helmholtz free energy contribution of the SRK equation of state, which contains the contribution of repulsive and dispersive interactions and *a* ^assoc^ is the contribution of association.

The SRK term can be expressed as^50^:

(4) $$a^{\mathrm{SRK}}=-\ln\left(1-\frac{b}{v}\right)-\frac{a\left(T\right)}{\mathrm{bRT}}\ln\left(1+\frac{b}{v}\right)$$

where *b* is the co‐volume parameter, *v* is the molar volume, and *a*(*T*) is the temperature dependent energy parameter of the mixture.

The *a* ^assoc^ is the same as that of PC‐SAFT (Equation (2)).

For the application of this equation of state five pure‐component parameters need to be specified for each associating fluid (or three for non‐associating fluids). These parameters are typically adjusted simultaneously to vapor pressure and saturated liquid density data. More information on the CPA equation of state are available from Kontogeorgis et al.^49^ Parameter sets for CPA used in this work along with a short description of each parameter can be found in Table S2 of Supplementary Material.

#### 2.1.3 Bonded fractions and average number of hydrogen bonds

An important structural property for associating compounds like water is the *k*‐times bonded fractions. In the SAFT framework, it is common to assume equivalence of sites. In other words, it is assumed that each site has the same probability to form a hydrogen bond. The maximum number of hydrogen bonds a water molecule can have is considered to be 4. Thus, due to the equivalence of sites, the *k*‐times bonded fractions *X*_*k*_ for a molecule that can form up to four hydrogen bonds can be expressed by considering a binomial distribution:

(5) $$X_{k}=\frac{4!}{\left(4-k\right)!k!}X_{A}^{\left(4-k\right)}{\left(1-X_{A}\right)}^{k}$$

where *X*_*A*_ is the fraction of molecules not bonded to site A. For *k* = 0, 1, 2, 3, 4, Equation (5) becomes:

(6) $$\begin{array}{l} X_{4}={\left(1-X_{A}\right)}^{4}\\ X_{3}=4{\left(1-X_{A}\right)}^{3}X_{A}\\ X_{2}=6{\left(1-X_{A}\right)}^{2}{X_{A}}^{2}\\ X_{1}=4{\left(1-X_{A}\right)}^{1}{X_{A}}^{3}\\ X_{0}={X_{A}}^{4} \end{array}$$

The thermodynamic models do not have the same amount of structural detail as molecular simulation models. For this reason, it is not possible to determine the fraction of molecules that are tetrahedrally coordinated without further assumptions. In this study, we are going to consider that the fraction of the molecules that form four hydrogen bonds (*X*_4_) is the same as the fraction of molecules that are tetrahedrally coordinated. Thus, we consider that *X*_4_ is equal to the LDL fraction since many authors consider LDL to be tetrahedrally coordinated.^11^, ^51^

Another property is the average number of hydrogen bonds *n*_HB_. By taking into account also the bonded fractions, *n*_HB_ can be expressed as:

(7) $$n_{\mathrm{HB}}=\sum_{k}{\mathrm{kX}}_{k}$$

When there is equivalence of sites and the set of Equation (6) is valid, then:

(8) $$n_{\mathrm{HB}}=4\left(1-X_{A}\right)$$

#### 2.1.4 Two‐state models for water

The theory that water consists of two different states is advocated by many scientists.^26^ There are some noteworthy models that view water as a “mixture” of two states (named as A and B), such as the ones developed by Holten et al.^52^ and by Caupin and Anisimov^53^ These models will also be used to some extent for our analysis. These models share a lot of similarities. Both are semi‐empirical and they consider a fluid with chemical equilibrium between two distinct inter‐convertible states. It needs to be underlined that this “mixture” is not a classical mixture of two different chemical species.^54^ These two states refer to the same chemical species (H_2_O), but they could have different arrangements in space. However, the models lack microscopic details and there are no structural information about the two states within the model. The key difference between these two states are their thermodynamic properties. For instance, the key equation of these two‐state models is the specific Gibbs free energy of the “solution,” which is expressed as:

(9) $$\frac{G}{R_{\mathrm{ig}}T}=\frac{G^{A}}{R_{\mathrm{ig}}T}+x\ln K_{\mathrm{eq}}+x\ln x+\left(1-x\right)\ln\left(1-x\right)+\mathrm{\omega x}\left(1-x\right)$$

where *G* is the specific Gibbs free energy of the “solution,” *G* ^*A*^ is the specific Gibbs free energy of state A, *x* is the “mole fraction” of state B in the fluid, *K*_eq_ is the chemical equilibrium constant of the “reaction” A⇄B, and *ω* is the interaction parameter. To evaluate the *G* ^*A*^, *K*_eq_, and *ω*, empirical expressions are used that are usually polynomials of temperature and pressure. Holten et al.^52^ and Caupin et al.^53^ have employed significantly different empirical expressions.

All of the thermodynamic properties are calculated at chemical equilibrium. The condition for chemical equilibrium is $\left(\frac{\partial G}{\partial x}\right)=0$ which results in the equation:

(10) $$\ln K_{\mathrm{eq}}+\ln\frac{x_{\mathrm{eq}}}{1-x_{\mathrm{eq}}}+\omega \left(1-2x_{\mathrm{eq}}\right)=0$$

where *x*_eq_ is the mole fraction of state B, that is, the LDL fraction, in chemical equilibrium and it is estimated by solving Equation (10).

Many of the parameters have been adjusted to experimental data of various thermodynamic properties of cold and supercooled water. More information about the two‐state models and the parameters can be found in the original publications.^52^, ^53^ For simplicity, the two‐state (TS) model of Holten et al.^52^ will be referred as TS/Holten and the version published by Caupin and Anisimov^53^ as TS/Caupin.

#### 2.1.5 Simulation methodology

We carried out molecular simulations with the inexpensive‐AMOEBA (iAMOEBA) water model.^55^ The iAMOEBA water model belongs to the Atomic Multipole Optimized Energetics for Biomolecular Applications (AMOEBA) class of molecular models. This water model has been parameterized using both experimental and ab initio data.^55^ It is a polarizable water model, but is less computationally expensive compared with its base AMOEBA model, because iAMOEBA only includes contributions to the polarization terms from the permanent fields. The remaining mutual polarization parameters have been optimized to reproduce both ab initio and selected experimental data.^56^ This water model has been reviewed extensively, and compared with many other water models in their ability to capture the structure and properties of water. It is capable of reproducing liquid‐phase properties of water like density, dielectric constant, self‐diffusion coefficient, and vapor–liquid equilibrium curve.^12^, ^56^, ^57^, ^58^ The iAMOEBA water model has also been used extensively in exploring the two‐state theory of water.^12^, ^59^ Utilizing its good accuracy in capturing the properties and structure of water, we recently employed the iAMOEBA water model to study the local tetrahedral environments in water, using a new structural descriptor.^60^ NPT simulations were carried out with a system of 2094 molecules, generating trajectories ranging from 10 to 100 ns. OpenMM 7.5^61^ was used to run the simulations. The length of simulations were dependent on the temperature—longer simulations were used at lower temperatures. Langevin leap‐frog integrator^62^ with a time‐step of 1 fs was used to perform the time integration. Pressure was maintained in simulations using the Monte‐Carlo barostat,^63^, ^64^ with a coupling time of 25 timesteps. The choice of the barostat and its coupling time has been done following a seminal work reported by Pathak et al. using the iAMOEBA water model.^12^ The simulation trajectories generated were read and analyzed using the MDAnalysis library.^65^

#### 2.1.6 Identifying hydrogen bonds

Hydrogen bonds are not declared a priori in simulations as a part of the force‐field, but are often assigned between two molecules in the trajectory if they satisfy certain criteria.^66^ These criteria are based on the geometry of the mutual orientation between the molecules in question and/or the energy of interaction between them.^67^ Recently we laid out a method to use the potential of mean force (PMF) landscapes to identify statistically favorable configurations in simulations, and use these configurations to define hydrogen bonds.^66^ Two molecules are said to be hydrogen bonded if their corresponding O–H distance (*r*) and the O–H–O angle (*α*) lie within the region of the PMF landscape defined by PMF ≤0 kT. Here, kT is the magnitude of thermal fluctuations in the system at temperature *T*. We reported that this region could be well captured by a partial ellipse.^66^

This method is also capable of identifying hydrogen bond configurations that lie deeper inside the PMF wells. We showed that hydrogen bonds which lie deeper inside the PMF well (PMF ≤ −2 kT) exhibit geometries similar to that of ice I_h_ (the hexagonal crystalline form of ice). Hydrogen bonds defined by this criterion were also observed^66^ to show statistics similar to that of strong hydrogen bonds reported by Wernet et al.^9^ Therefore, hydrogen bonds that lie in the region PMF ≤ −2 kT were distinguished as “ice‐like” or “strong” hydrogen bonds.^66^ Interested readers are referred to our previous work for more details.^66^

#### 2.1.7 Estimation of LDL fraction from O–O–O angles

We recently showed^60^ that the structure of tetrahedral environments could be identified based on the average angle an oxygen atom makes with its neighbors (*θ*_avg_). The distribution of *θ*_avg_ is bimodal with one peak at around 109.5°, which is the internal angle of a regular tetrahedron. This indicates the presence of two structural environments, one of which resembles the structure of a tetrahedron. The probability distribution of *θ*_avg_ was decomposed into two constituent skewed Gaussian distributions (Equation (11)). Here, subscript *s* indicates the tetrahedral population and *ρ* indicates the non‐tetrahedral population.

(11) $$P\left(\theta \right)=s\times G_{\text{skew}}\left(\theta ,\mu_{s},\sigma_{s};\alpha_{s}\right)+\left(1-s\right)\times G_{\text{skew}}\left(\theta ,\mu_{\rho },\sigma_{\rho };\alpha_{\rho }\right)$$

In Equation (11), *s* is the fraction of tetrahedral population in liquid water, and *μ*, *σ*, and *α* are, respectively, the mean, standard deviation, and skewness of the individual Gaussian distribution. The subscripts to these parameters indicate the respective populations. The individual Gaussian distributions are given by Equation (12).

(12) $$G_{\text{skew}}\left(\theta ;\mu ,\sigma ,\alpha \right)=\frac{1}{\sigma \sqrt{2\pi }}\exp\left(-\frac{\theta -\mu }{2\sigma^{2}}\right)\times \left[1+\mathrm{erf}\left(\frac{\alpha \left(\theta -\mu \right)}{\sigma \sqrt{2}}\right)\right]$$

where, erf(*x*) is the error function given by Equation (13).

(13) $$\mathrm{erf}\left(x\right)=\frac{2}{\sqrt{\pi }}\int_{0}^{x}e^{-t^{2}}\mathrm{dt}$$

The parameters of Equation (11) were estimated by fitting the probability distribution of *θ*_avg_ from molecular simulations, and minimizing the least square of deviation from the right hand side of Equation (11). The parameter *s* gives the estimate of the LDL fraction in liquid water.

## 3 RESULTS AND DISCUSSION

**FIGURE 1:**
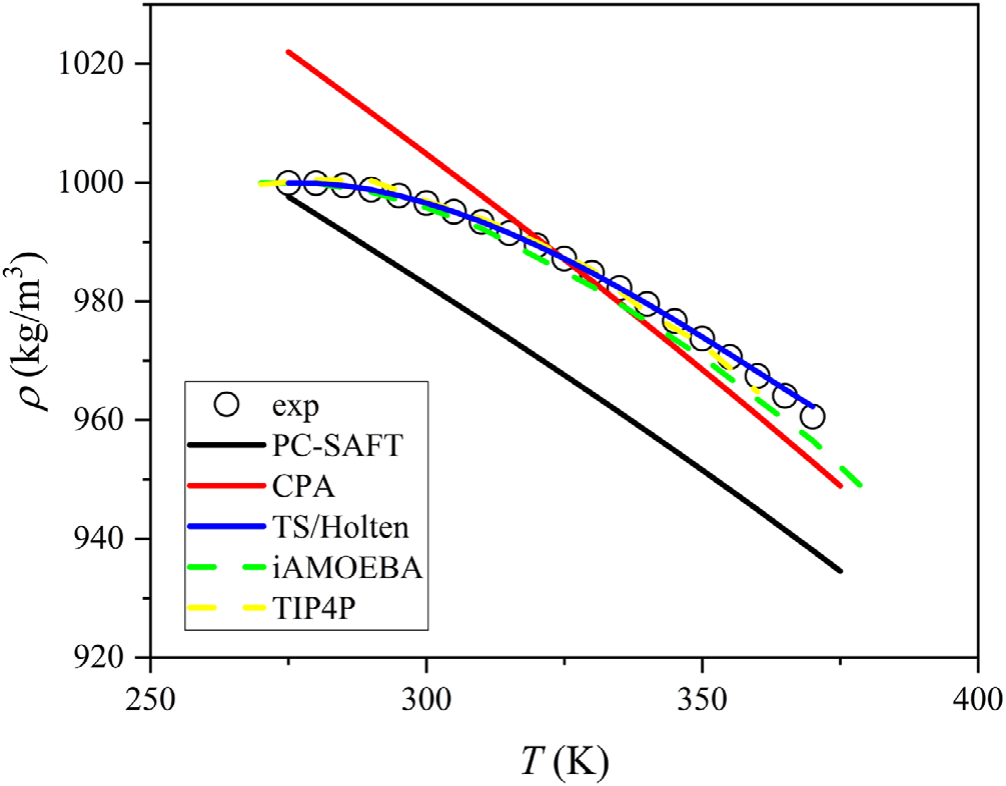
Density of liquid water at 1 bar. Symbols are correlations from NIST^68^ and lines are results from the models. Dashed lines are our results using molecular simulation models (iAMOEBA^55^ and TIP4P/2005^69^). Solid lines are our results with thermodynamic models (TS/Holten,^52^ PC‐SAFT with the parameter set P1^46^ and CPA^70^).

In Figure 1, the density of liquid water at 1 bar is shown calculated with different models. PC‐SAFT and CPA are not able to predict the density anomaly. In fact, these two models are not able to predict any of water's anomalies.^3^ On the other hand, TIP4P/2005^69^ and iAMOEBA^55^ are able to predict a density maximum and they predict water's density almost perfectly. In addition, the TS/Holten model^52^ is able to capture the density maximum. The reason why some models are able to predict the density anomaly while others cannot is likely connected to the models' description of water's structure. The TS/Holten is based on the two‐state hypothesis and molecular simulation models (TP4P/2005 and iAMOEBA) also consider that polar interactions can alter the structure, which may result in two distinct structures. PC‐SAFT and CPA do not incorporate the two‐state hypothesis in any way.

**FIGURE 2:**
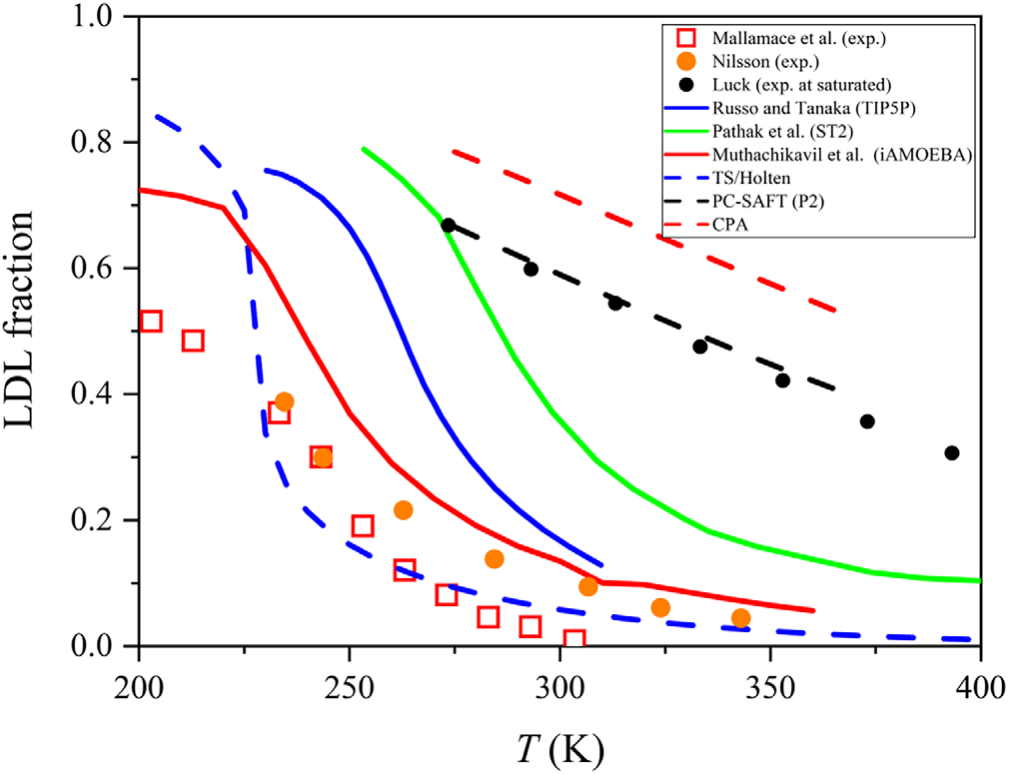
LDL fraction of water from different sources (most at 0.1 MPa). Solid lines are estimations from molecular simulations (Russo and Tanaka,^51^ Pathak et al.,^12^ and Muthachikavil et al.^60^) and symbols are data estimated from spectroscopy techniques (Luck,^5^ Mallamace et al.,^71^ and Nilsson^21^) and dashed lines are our estimations from SAFT‐type models (PC‐SAFT parameter set of Haghmoradi et al.,^72^ CPA parameter set from Kontogeorgis et al.^70^) and the model TS/Holten.^52^ Luck's data are a saturated conditions. An overview of the data and model estimations can be found in the section S1 of Supplementary Material “Brief overview of LDL fraction data.” In this section, it is also discussed how Luck's free OH group data were used to estimate the LDL fraction.

Figure 2 shows LDL fraction values from simulations,^12^, ^51^, ^60^, ^73^ two‐state model,^52^ spectroscopy data^5^, ^21^ and from thermodynamic model predictions.^7^, ^46^, ^70^, ^72^ There is scatter from the various sources (see also Figure S1 of Supplementary Material). Most of the estimates tend to show similar sigmoid trends, with the exception of the association models (PC‐SAFT and CPA) and Luck's data (at saturation). The data of Mallamace et al.^71^ show the lowest LDL fractions, not very far from two‐state model calculations and the recent experimental data from Nilsson.^21^ Luck's earlier measurements show much higher LDL. Thermodynamic model predictions from the association theories show the highest LDL fractions, indicating a much higher tetrahedral fraction, in agreement with the incorporated tetrahedral structure in these models. For the thermodynamic models, it is assumed that molecules with four hydrogen bonds follow tetrahedral coordination. Moreover, although these models and Luck's data also show a decreasing tetrahedral fraction with increasing temperature, the trend is largely linear, unlike the trend seen from the other LDL sources. To the best of our knowledge, no effort has been made in literature to incorporate the LDL trends from two‐state theories into the advanced thermodynamic models.

**FIGURE 3:**
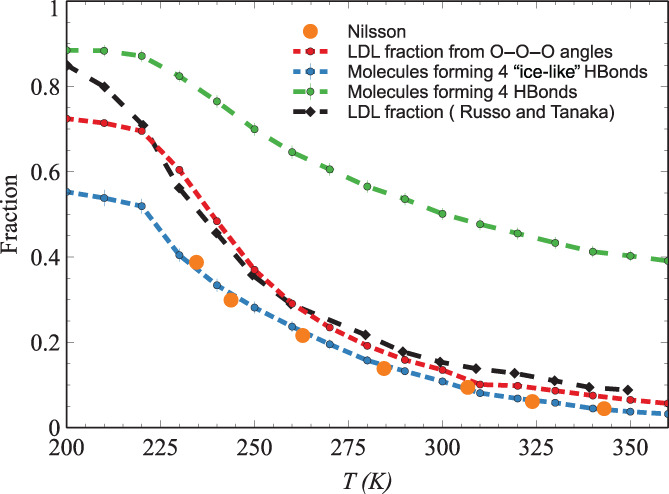
The most recent LDL fraction data recommended by A. Nilsson^21^ compared with the values estimated from O–O–O angles,^60^ the fraction of molecules forming 4 hydrogen bonds,^66^ the fraction of molecules forming 4 “ice‐like” hydrogen bonds,^66^ and the LDL fraction reported by Russo and Tanaka.^51^ All the values shown are estimated from simulations at a pressure of 1 bar.

Figure 3 shows another aspect of our analysis: LDL from molecular simulation studies as compared with the most recent data recommended by Nilsson.^21^ We can see that there is a very good agreement between LDL fraction from literature and molecular simulations, especially when only “ice‐like” hydrogen bonds^66^ are considered. On the other hand, it can also be seen that the fraction of molecules forming four hydrogen bonds (when all hydrogen bonds are considered) grossly over estimates the fraction of local tetrahedral structures in liquid water. The comparison in Figure 3 demonstrates the relevance of characterizing hydrogen bonds in liquid water, in the analysis of local tetrahedral structures in water.

**FIGURE 4:**
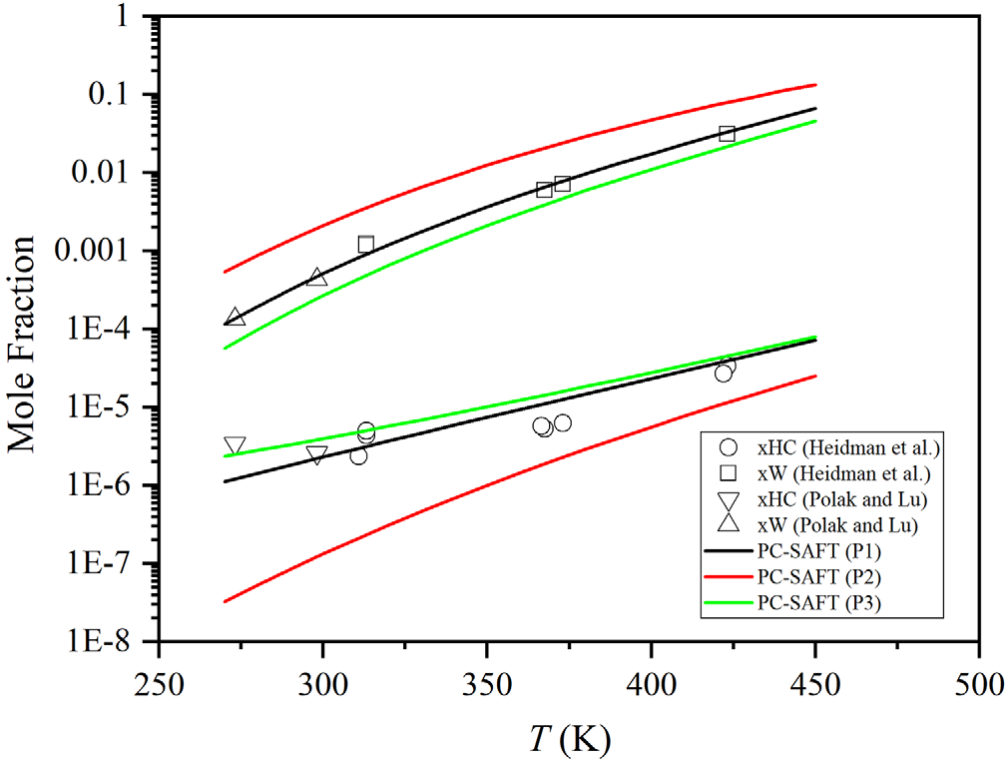
Mutual solubilities of water and n‐hexane. Symbols refer to experimental data of Heidman et al.^74^ and of Polak and Lu.^75^ xHC: solubility of n‐hexane in water. xW: solubility of water in n‐hexane. Solid lines are estimations from PC‐SAFT with three different parameter sets (P1,^46^ P2,^72^ and P3^47^). For all calculations, we have considered the interaction parameter to be equal to zero. More information about the parameter sets P1, P2, and P3 can be found in the Section S5 of Supplementary Material.

Efforts have been made to use or represent the Luck's experimental data and the PC‐SAFT version of Haghmoradi et al.^72^ (set P2) represents well these data, as seen in Figure 2. This is done at enormous cost for other properties as seen in Figure 4, illustrating that this model with such parameters cannot predict the liquid–liquid equilibria of water–alkanes satisfactorily, unlike the other parameter sets (P1^46^ and P3^47^). From Figure S2 of Supplementary Material, one can notice that even with an interaction parameter, the adjusted performance is not improved with these parameters. Other versions of the same model with different parameters (such as P1 and P3) represent liquid–liquid equilibria very well. We have made similar observations before.^46^ It seems that it is possible to reproduce monomer fraction data for water with SAFT‐type models at the cost of representing liquid–liquid equilibria for water–hydrocarbons. Figure 4 demonstrates another characteristic of aqueous mixtures, which cannot be captured by advanced thermodynamic models without additional parameters: the minimum of the hydrocarbon solubility in water, which is often associated with the hydrophobic effect.

**FIGURE 5:**
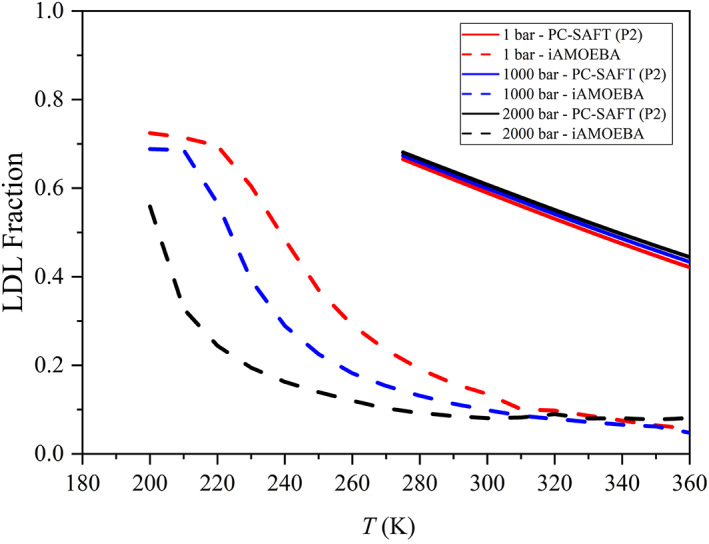
LDL fraction isobars estimated using different methods. Solid lines are our estimations from PC‐SAFT with the parameter set P2^72^ and dashed lines are from simulations with the iAMOEBA water model.^60^

Figure 5 illustrates a further deficiency of the advanced models when the LDL fraction is plotted as functions of temperature and pressure. Here, the LDL fraction estimates from PC‐SAFT using the P2 parameter set^72^ and our estimates with iAMOEBA are shown and compared. Estimates from iAMOEBA show a sigmoid trend at all pressures, while PC‐SAFT estimates show an almost linear trend at all pressures. Moreover, LDL fractions from iAMOEBA are decreasing when pressure increases, while LDL fractions predicted by PC‐SAFT slightly increase with an increase in pressure. It should be noted that this pressure dependence of LDL fractions is not only captured by iAMOEBA, but there are others that have shown similar trends^12^, ^53^ (also displayed in Figures S3 and S4). Only PC‐SAFT is predicting linear trends in the LDL fraction and at the same time only PC‐SAFT is unable to predict any of water's anomalies. It is likely that the LDL fraction and the anomalous properties of water are connected.

**FIGURE 6:**
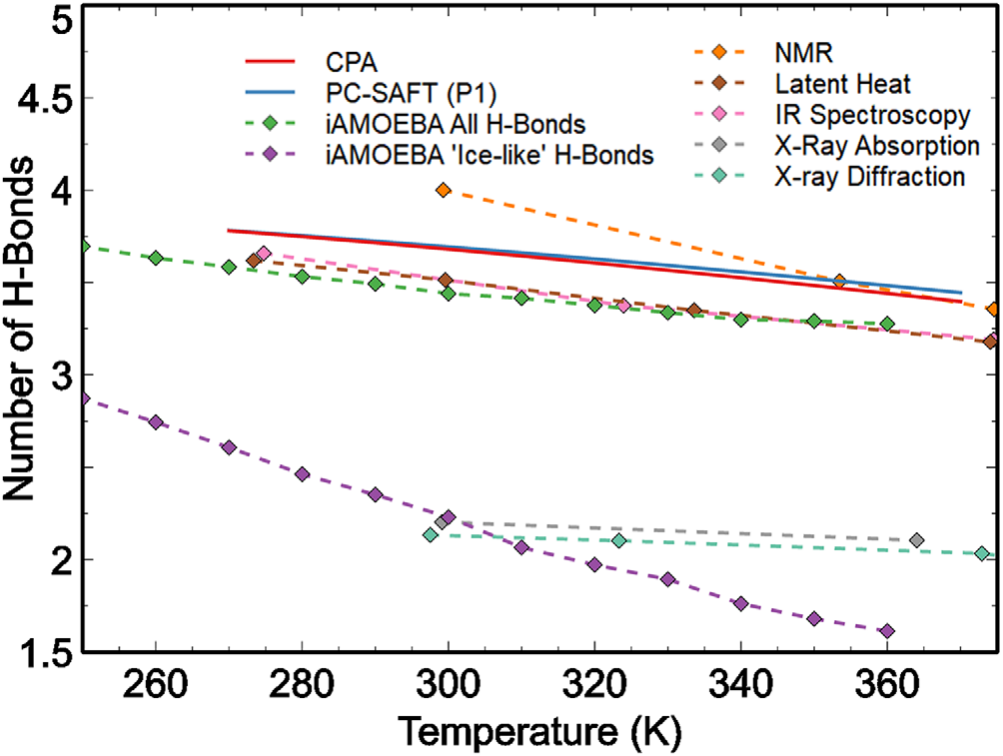
Variation of the average number of hydrogen bonds estimated from thermodynamic models (CPA,^70^ PC‐SAFT (P1),^46^), experiments (NMR,^76^ latent heat,^77^ IR spectroscopy,^5^ X‐ray absorption,^9^ pair correlation functions from X‐Ray diffraction^78^), and molecular simulations (of iAMOEBA water model).^66^ The results from CPA and PC‐SAFT (P1) are calculated using Equation (8).

An important property, which is related to the LDL fraction and the site fraction, is the average number of hydrogen bonds (*n*_HB_) shown in Figure 6. Different experimental approaches, theories and computer simulations have been employed in literature to estimate the average number of hydrogen bonds formed by a water molecule.^5^, ^9^, ^46^, ^66^, ^70^, ^76^, ^77^ Figure 6 shows the estimates from some of these studies. The general understanding has been that most water molecules form four hydrogen bonds. Two of which are through donating the slightly positive hydrogen atoms and the other two are by accepting two hydrogen atoms from neighboring molecules.^29^ This leads to an average number of hydrogen bonds slightly less than 4, as confirmed by different thermodynamic models,^46^, ^70^ simulations^66^ and experimental methods like nuclear magnetic resonance (NMR),^76^ latent heat measurements,^77^ and infrared (IR) spectroscopy.^5^ There are still deviations from the number reported using different methods. However, results published by Wernet et al.^9^ using X‐ray absorption suggested that most water molecules form only 2 strong hydrogen bonds—by donating one hydrogen atom and by accepting another hydrogen atom from a neighbor. This resulted to an estimated average of about 2.2 hydrogen bonds per molecule.^9^ We recently demonstrated that “strong” or “ice‐like” hydrogen bonds may be identified in simulations based on the PMF landscapes.^66^ This definition of “strong” hydrogen bonds also indicate that most water molecules form only 2 strong hydrogen bonds, agreeing with the statistics reported by Wernet et al.^9^ Results reported by Gorbaty et al. using pair correlation functions estimated from X‐ray diffraction also suggest that the average number of hydrogen bonds formed by a water molecule is about 2 at room temperatures.^78^ In spite of the disagreements between the actual number of hydrogen bonds formed by a water molecule, there is an agreement regarding the temperature trend of the number of hydrogen bonds. At higher temperatures, higher magnitudes thermal fluctuations can break hydrogen bonds more easily. As can be seen from Figure 6, this leads to a downward trend of the average number of hydrogen bonds. We observe that there is a significant variation from the various sources but in all cases *n*_HB_ decreases with increasing temperature. This decrease is more modest from association theories compared with the values obtained from the literature, which also indicates structural fluctuations (from LDL to HDL and vice versa).

**FIGURE 7:**
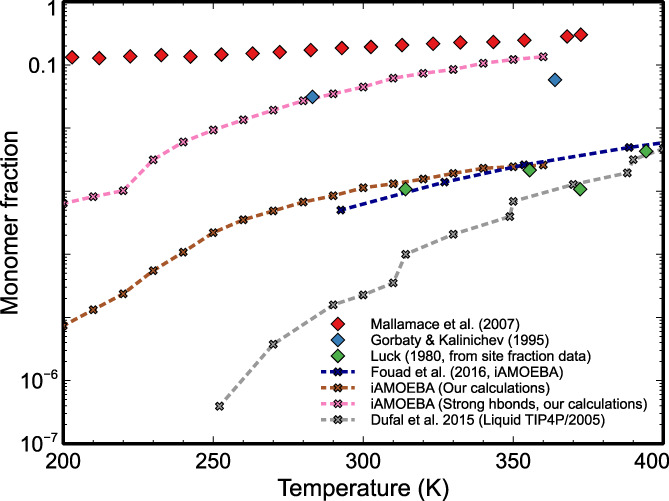
Monomer fractions of water as function of temperature from various sources. Experimental results reported by Mallamace,^71^ Gorbaty et al.,^79^ Luck,^5^ and simulation results reported by Fouad et al.,^80^ Muthachikavil et al. (iAMOEBA water model),^66^ and Dufal et al.^7^ at a pressure of 1 bar are compared.

Another source of structural information for water available from several types of measurements, simulations and models is the monomer fraction. Figure 7 shows simulations from literature,^80^ measurements from Mallamace et al.,^71^ Luck,^5^ and Gorbaty and Kalinichev^79^ and our simulations. Fouad et al.^80^ used two different water models (TIP4P/2005 and iAMOEBA) and we have used the latter force field.

Fouad et al. defined hydrogen bonds based on geometric criteria, with a rectangular cut off (O–O distance ≤3.7 Å and O–O–H angle ≤30°). The monomer fractions were then calculated as the fraction of molecules that do not form any hydrogen bond with their neighbors. Luck's estimates from spectroscopy was the fraction of free OH groups (free site fractions). The monomer fractions were then estimated based on the free site fractions using set of Equations (6).

The earlier studies by Gorbaty and Kalinichev^79^ used spectroscopy in supercritical water. They reported that hydrogen bonds do not disappear in water at temperatures as high as 800 K. The fractions of monomers reported by Gorbaty and Kalinichev are higher than those reported by Luck.

Mallamace et al.^71^ used spectroscopy techniques to estimate the fraction of molecules forming different number of hydrogen bonds. They attributed different peaks of the spectra to different structural entities like fully hydrogen bonded (four hydrogen bonds), partially hydrogen bonded (1, 2, or 3 hydrogen bonds) or non‐hydrogen bonded (zero hydrogen bonds). The fraction of non‐hydrogen bonded molecules, which is essentially the fraction of free monomers, is also shown in Figure 7. The estimates by Mallamace et al.^71^ in this work are much higher than all other estimates. Since Mallamace et al. based their work on identifying hydrogen bonded structures in relation to the structure of low density amorphous ice, we compared the fraction of monomers, as per the “ice‐like” hydrogen bonds with their monomer fractions. However, the results reported by Mallamace et al. are observed to be even higher than the monomers counted in our simulations when considering only “ice‐like” hydrogen bonds. We can see that Fouad et al.'s simulations with iAMOEBA^80^ are in good agreement with the data based on Luck's experiments.^5^ They also showed that the monomer fractions estimated from iAMOEBA were closer to Luck's results (when compared with TIP4P/2005). However, they also concluded that there is a deviation between the monomer fraction predicted by both iAMOEBA and Luck, with the estimates from the polar PC‐SAFT model (an advanced model which includes an additional polar term). The estimates of Dufal et al. (using TIP4P/2005) water model also shows a similar temperature trend for monomer fractions, but the values are smaller than that of the iAMOEBA water model.^7^ Estimates from our simulations of the iAMOEBA water model (defined as the fraction of molecules which are not hydrogen bonded to any of its neighbors) are also shown in Figure 7. We defined hydrogen bonds based on an elliptical cut‐off based on the PMF landscape.^66^ Our studies were focused more on the supercooled regimes of water, but in the temperatures common with Fouad's calculations, we find similar results. The molecules forming no hydrogen bonds essentially point to free monomers in water. These results shown in Figure 7 illustrate good similarity with Luck's monomer fraction data and our own estimates from simulations of the iAMOEBA water model.

**FIGURE 8:**
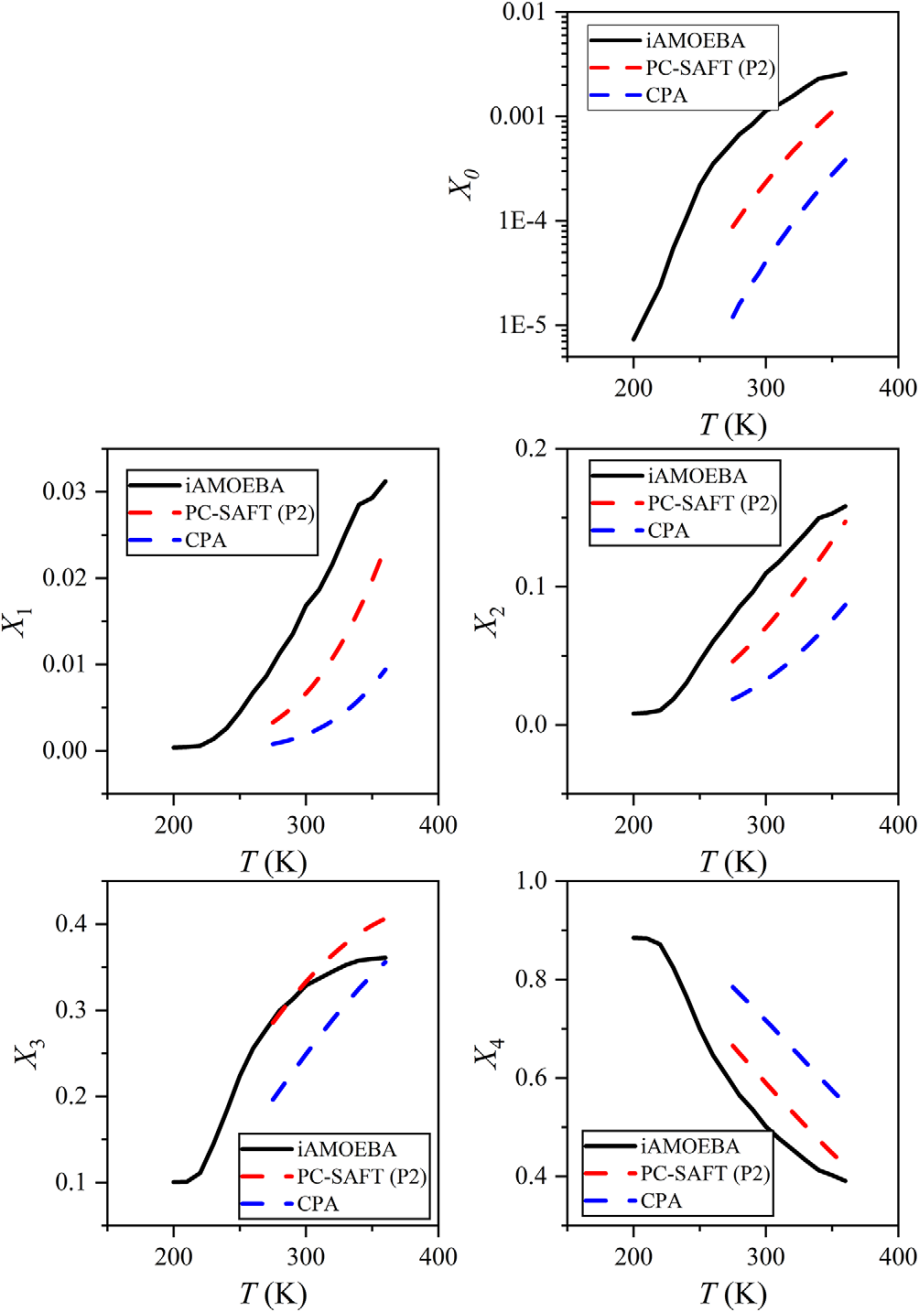
Fraction of water molecules (in liquid water) bonded *k*‐times (*k* = 0–4) from our simulations using the iAMOEBA water model, our calculations PC‐SAFT (with set P2)^72^ and CPA.^70^ All these calculations refer to 1 bar.

In the results presented so far we have considered, from experimental data, two‐state theories, simulations and thermodynamic models, only the LDL (tetrahedral) fraction and monomers. It is clear that advanced thermodynamic models (PC‐SAFT and CPA) have shown considerable differences for these structural properties, especially for the LDL fraction. These are not the only structural properties that can be considered. These thermodynamic models and molecular simulations can offer information about fraction of molecules bonded *k*‐times (*k* ∈ 0, 1, …, 4). These fractions can offer a more complete description about the degree of hydrogen bonding in the system. In Figure 8 we have compared the fractions of *k*‐times bonded molecules at 1 bar with iAMOEBA, PC‐SAFT, and CPA. Again, there is a clear quantitative difference for most of the bonded fractions, even though interestingly enough the set P2 has a near excellent agreement with iAMOEBA for *X*_3_ at certain conditions. Additionally, in Supplementary Material 1 there are more comparisons of *k*‐times bonded fractions with published data from Fouad et al.^80^ that refer to saturated conditions (Figures S5 and S6).

These results, in addition to results from Figures 2, 3, 5, and 6 indicate that modern thermodynamic models do not reflect the structure of water accurately since there are considerable differences to the degree of hydrogen‐bonding. This can be related to structural fluctuation which are believed to be caused by local structures that are characterized by different hydrogen bonded networks.^11^, ^51^, ^60^

## 4 CONCLUSIONS

Advanced thermodynamic models incorporate water's hydrogen bonding using the tetrahedral structure. They have shown much greater accuracy for describing pure water's properties and phase equilibria compared with “classical” models, but they are still unable to predict water's anomalous behavior. An immediate conclusion is that the origin of these anomalies is not included in the models. There have been attempts to modify the models, either by including co‐operativity^72^, ^81^ or by including structural changes in the reference fluid.^4^ The latter has been successful at describing some of water's anomalies, like the density maximum, but results are still very preliminary.^4^

For the classical association theories such as the ones we consider in this work, we observe a qualitative only agreement against the Luck's data and much higher LDL fractions compared with those from experimental data and two‐state theories. In the early days of the developments of the association models (1980–2000), the only structural data available for comparisons were those from the spectroscopic studies of Luck and also from Gorbaty and a few other researchers. Later some simulation data became available (while the interpretation‐definition of hydrogen bonds remains a serious issue). Luck's and Gorbaty's data do show lower hydrogen bonding than the association thermodynamic models but the community was, possibly, less concerned at that time. This is because association theories performed well for phase equilibria and such spectroscopic data are both rare and maybe not so accurate. Some other considerations, for example, theories which connected site fractions to dielectric constants^82^ agreed more with the thermodynamic models rather than the spectroscopic data.^6^ So, there was a feeling that Luck's and other data might be more in error rather than the theories.

In this study we have considered recent data from simulation, experiments, and two‐state theories. The underlying conclusion is that the hydrogen bonding of water molecules leading to tetrahedral structures must be much smaller than what previously anticipated, even lower than Luck's and Gorbaty's data and, thus, in complete disagreement even when compared with the “best” association theories.

It is too early to conclude that the representation of anomalous properties of water can only be represented by a consideration of a very low LDL (tetrahedral fraction), as indicated from the two‐state theories and associated experimental measurements, or even by considering the two‐state concept in its totality. The work of Holten et al.,^52^ where two‐state theory is implemented in a thermodynamic context, is of relevance. But in Holten et al.,^52^ no structures are assumed for LDL and HDL, there are multiple parameters and very poor results are obtained outside the range in which the parameters have been estimated.^83^ This model is semi‐empirical and it seems that it relies heavily on adjustable parameters and empirical terms. Possibly for this reason the model provides unrealistic results outside the range of parameter estimation. If the parameters were to be readjusted to data that refer to high temperatures as well, then maybe the model would perform much better at these conditions.

The results of this study clearly point out on a connection between the failure of advanced association thermodynamic models for anomalous properties and water structure and the presence of a very low LDL fraction. This study indicates that intense structural changes (fluctuations) lead to water's anomalies, which are present in molecular simulations and two‐state models that are able to capture water's anomalies, but these structural changes are absent in thermodynamic models. Nonetheless, it is always important to recognize that simulations as well as thermodynamic models may never be fully in agreement with experimental results. However, it is important to recognize what aspect of models needs to be improved, so that we can approach experimental results closer. In the case of water, our analysis shows that it is the aspect of structural fluctuations that is missing in modern thermodynamic models, and in order to predict experimental results closely, it is a very important aspect that needs to be included.

## AUTHOR CONTRIBUTIONS

**Evangelos Tsochantaris:** Conceptualization (equal); data curation (lead); formal analysis (lead); methodology (equal); software (lead); validation (lead); visualization (lead); writing – original draft (lead); writing – review and editing (equal). **Aswin V. Muthachikavil:** Conceptualization (equal); data curation (lead); formal analysis (lead); methodology (equal); software (lead); validation (lead); visualization (lead); writing – original draft (lead); writing – review and editing (equal). **Baoliang Peng:** Conceptualization (equal); funding acquisition (lead); project administration (equal); resources (equal); writing – review and editing (equal). **Xiaodong Liang:** Conceptualization (lead); funding acquisition (equal); methodology (lead); project administration (lead); resources (lead); supervision (lead); writing – review and editing (lead). **Georgios M. Kontogeorgis:** Conceptualization (lead); funding acquisition (lead); methodology (lead); project administration (equal); resources (lead); supervision (lead); writing – review and editing (lead).

## FUNDING INFORMATION

Horizon 2020 research and innovation program, ERC Advanced Grant Project “New Paradigm in Electrolyte Thermodynamics,” Grant Number: 832460; Scientific Research and Technology Development Project of RIPED, PetroChina, Grant/Award Number: YGJ2019‐11‐01.

## CONFLICT OF INTEREST

The authors declare no conflicts of interest.

## Supporting information


**Appendix S1** Supporting Information.

## Data Availability

Data available in article supplementary material.
