# Supplementary material for: Multiple insights call for revision of modern thermodynamic models to account for structural fluctuations in water
Source: AIChE J. 2022 Sep 5;68(11):e17891. doi: 10.1002/aic.17891 (PMC9787682; doi:10.1002/aic.17891)
Supplement: Supplementary file 1 — Appendix S1 Supporting Information. [file AIC-68-e17891-s001.pdf]

Supplementary Material for

Multiple Insights Call for Revision of Modern Thermodynamic Models to Account for Structural Fluctuations in Water

Evangelos Tsochantaris[1*], Aswin V. Muthachikavil[1*], Baoliang Peng[2], Xiaodong Liang[1,a] and Georgios M. Kontogeorgis[1,b]

1Center for Energy Resources Engineering, Department of Chemical and Biochemical Engineering, Technical University of Denmark, Kgs. Lyngby 2800, Denmark;
2Research Institute of Petroleum Exploration & Development (RIPED), PetroChina, Beijing 100083, China;
* Equally contributing authors
Corresponding Authors: Georgios M. Kontogeorgis and Xiaodong Liang
aEmail: xlia@kt.dtu.dk
bEmail: gk@kt.dtu.dk

S1 Brief overview of LDL fraction data

Figure 2 of the main article contains LDL (low-density liquid) fraction estimates from computational studies as well as from experimental studies. More data are presented below in Figure S1. In this section, there is a brief overview about the methods that have been used. Pathak et al. [1], and Russo and Tanaka [2] based their estimations on molecular simulations and they used the same method for identifying the LDL structure. The only difference was in the model where Pathak et al. [1] used ST2 model and Russo and Tanaka used TIP5P [2]. This suggests that the two models could have some differences at describing water's structure (i.e. different radial distribution function). Muthachikavil et al. [3] used molecular simulations of the iAMOEBA water model to identify local structures. They used a method similar to the one described by Russo and Tanaka [2] but the criterion for the two-states is different since it is defined by the average angels of oxygen atoms with all its neighbors. Moore and Molinero [4] found local structures in the mW model for water (molecular simulations) by trying to identify tetrahedral structures. Holten et al. [5] developed a two-state thermodynamic model for water, where the two hypothesized states are in chemical equilibrium. As it is mentioned in the paper it is a semi-empirical model with many adjustable parameters (around 77). Overall, computational studies suggest that water is at all times a mixture of the two different structures because of the asymptotes, but data of Mallamace et al. [6] suggest that LDL is non-existent or near non-existent after 300 K. Mallamace et al. [6] have published more LDL fraction data at even lower temperatures, but the estimates at these low temperatures were based from Raman spectroscopy on low-density amorphous ice. Nilsson [7] has used X-ray spectroscopy to estimate LDL fractions in cooled and mostly supercooled water. Nilsson's estimates are the most recent experimental ones to date. Some of the spectroscopy data that have considerable uncertainty values are summarized in Table S4.

Regarding the data of Luck [8] that are shown in Figure 2 of the main article, it should be mentioned that they refer to percentage of free OH groups, which is the same as the free site fraction X_A. These data were turned to the 4-times bonded fractions X_4 by assuming the equivalence of sites. In other words we used the expression $X_4 = (1 - X_A)^4$. For Luck's data and thermodynamic models, we consider that the fraction of molecules bonded 4 times X_4 is the LDL fraction (tetrahedral fraction). In other words, we assume that all molecules with 4 hydrogen bonds follow the tetrahedral structure. This assumption might be one of the reasons why LDL fractions of Luck and of thermodynamic models are in general higher compared to the rest.

S2 Mutual solubilities of water – n-hexane

In Figure 4 of the main article, mutual solubilities are shown of water and n-hexane. In Figure 4 (main ariticle), the interaction parameter k_{ij} was considered to be 0 and the model results are predictions (not fitted to solubility data). Usually, the interaction parameter is fitted so that the calculated solubility of n-hexane in water matches the actual values. The fitted k_{ij} values are shown in Table S5. Figure S2, shows results with fitted k_{ij} values. After the fitting, all of the models are able to describe the solubility of water in n-hexane. Most of the parameter sets show excellent accuracy for the solubility of n-hexane in water with the exception of the P2 parameter set which produces results several orders of magnitude below the actual values. However, none of the parameter sets is able to predict the minimum in the solubility of n-hexane in water .The parameter set of P2 is the most accurate at predicting Luck's data, but it is also by far the most inaccurate for predicting the solubility of water in n-hexane. The interaction parameter for interactions between molecule i and j has been used in a combining rule of PC-SAFT:

$$\varepsilon_{ij} = (1 - k_{ij})\sqrt{\varepsilon_{ii}\varepsilon_{jj}} \tag{1}$$

Where ε_{ij} is the dispersion energy parameter for the interaction between molecules i and j,and ε_{ii} and ε_{jj} are the dispersion energy parameters of molecule i and j, respectively.

S3 Deviations for bonded fractions

In Tables S6-S8, deviations are shown for Figure S6. The estimates for fraction X_1 seem to have the largest deviations when compared to TIP4P/2005 values from Fouad et al. [9]. To calculate the absolute average deviations we are using the following expression:

$$\%AAD(X) = \frac{1}{N_p}\sum_{i=1}^{N_p}\left|\frac{X_{calc,i} - X_{exp,i}}{X_{exp,i}}\right| \tag{2}$$

where $X_{calc,i}$ is any calculated fraction $(X_A, X_0, X_1, X_2, X_3, X_4)$ from the thermodynamic models for data point i and $X_{exp,i}$ is the corresponding fraction $(X_A, X_0, X_1, X_2, X_3, X_4)$ that has been published from Luck [8] or Fouad et al. [9] and N_p is the total number of published values for each property.

S4 Supplementary figures

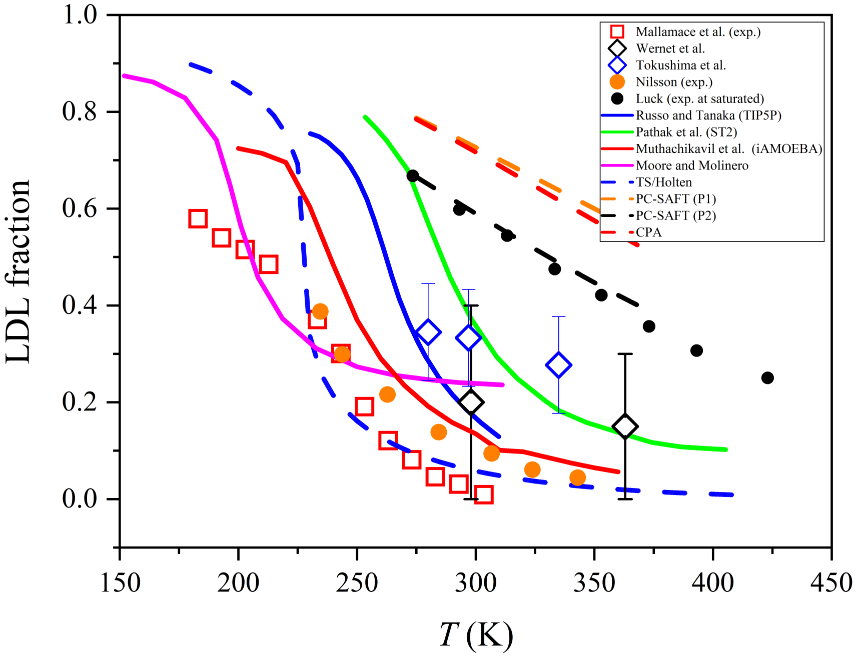

Figure S1: LDL fraction of water from different sources (most at 0.1 MPa). Solid lines are estimations from molecular simulations (Russo and Tanaka [2], Pathak et al. [1], Muthachikavil et al. [10], Moore and Molinero [4]) and from a two-state model (TS/Holten) [5], symbols are data estimated from spectroscopy techniques (Luck [8], Mallamace et al. [6], Nilsson [7], Tokushima et al. [11], Wernet et al. [12]) and dashed lines are estimations from thermodynamic models (PC-SAFT with the parameter set of Haghmoradi et al. [13], CPA with the parameter set from Kontogeorgis et al. [14] and two-state model [5]). Luck's data are in saturated conditions. Dashed lines are our calculations.

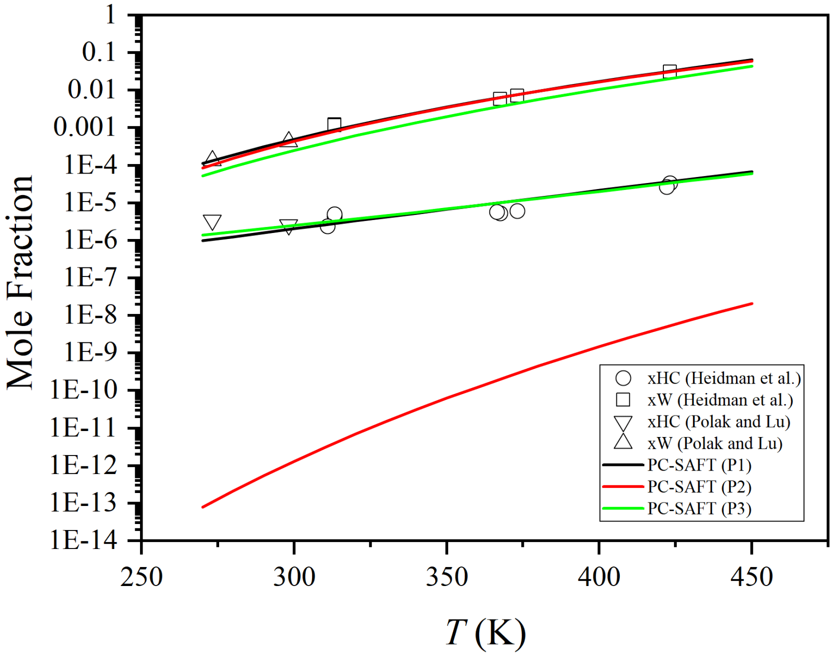

Figure S2: Mutual solubilities of water and n-hexane. Symbols refer to experimental data of Heidman et al. [15] and of Polak and Lu [16]. xHC : solubility of n-hexane in water. xW : solubility of water in n-hexane. Solid lines are estimations from PC-SAFT with three different parameter sets (P1: Liang et al. [17], P2: Haghmoradi et al. [13], P3: Diamantonis and Economou [18]). For all calculations, we have fitted the interaction parameter (k_{ij}) values to the soulubility oh n-hexane in water (xHC). Information about the thermodynamic models, the parameter sets and calculation can be found in the section "Thermodynamic models".

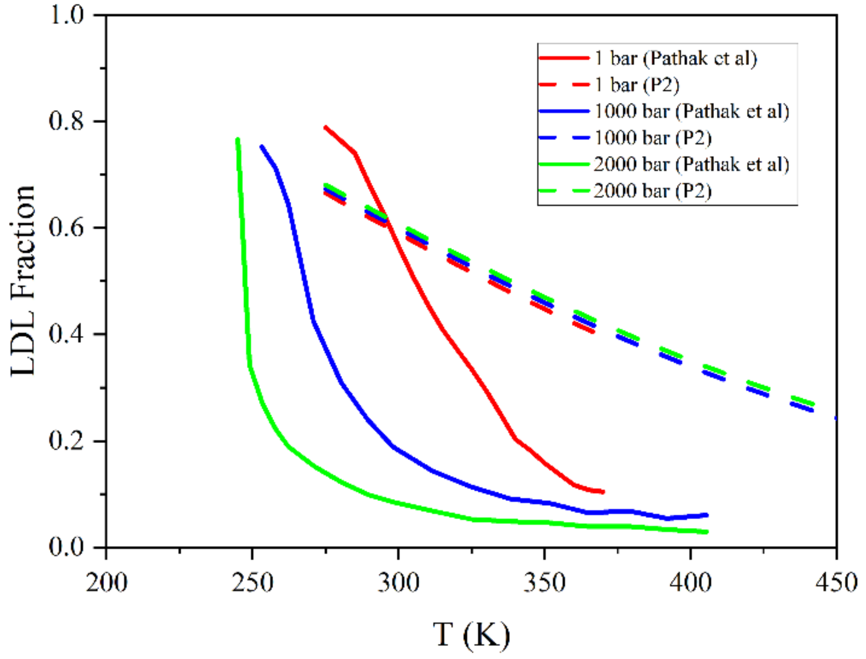

Figure S3: LDL fraction isobars. Solid lines are published estimates of Pathak et al. [1] at different pressures using the ST2 model of water and dashed lines are estimations from PC-SAFT with the parameter set P2 [13].

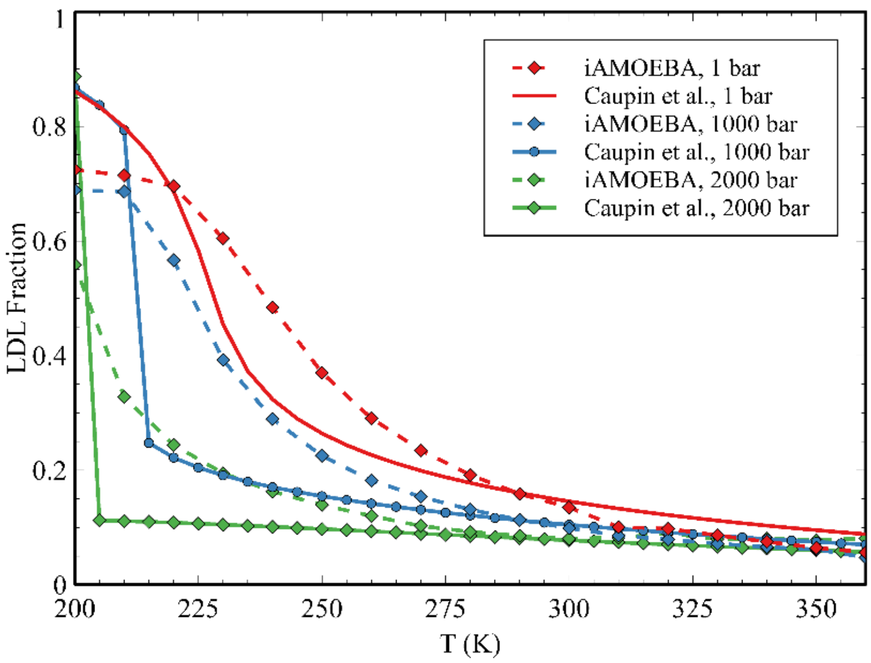

Figure S4: LDL fraction isobars. Solid lines are published estimates from the model of Caupin et al. [19] and dashed dotted lines are estimates from iAMOEBA (estimated using O-O-O angles [10]).

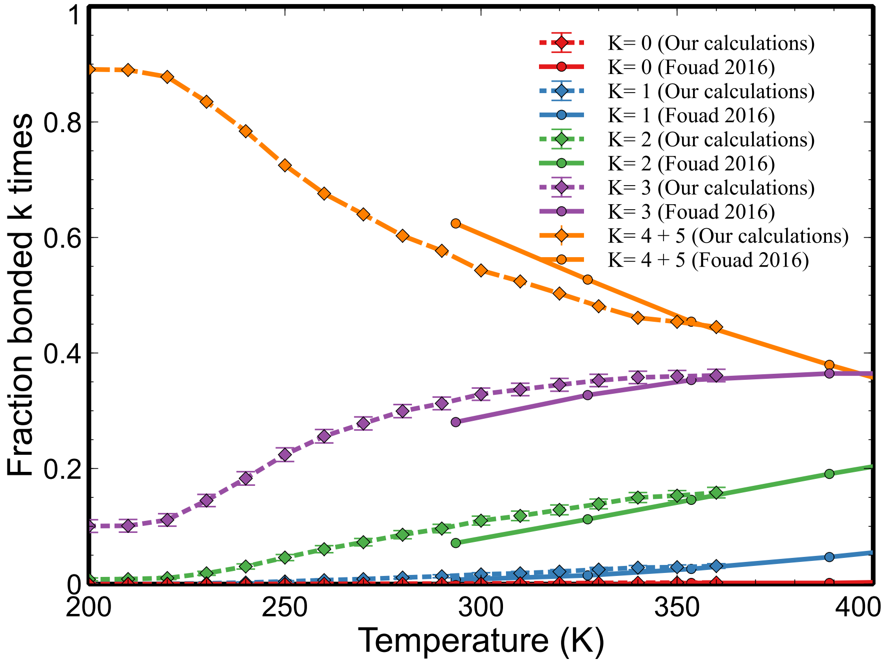

Figure S5: Fraction of water molecules (in liquid water) bonded k-times (k=0-4) from our simulations (at 1 bar) using the iAMOEBA water model and the values reported by Fouad et al [9]. The values reported by Fouad et al are using iAMOEBA water model, but at saturation conditions.

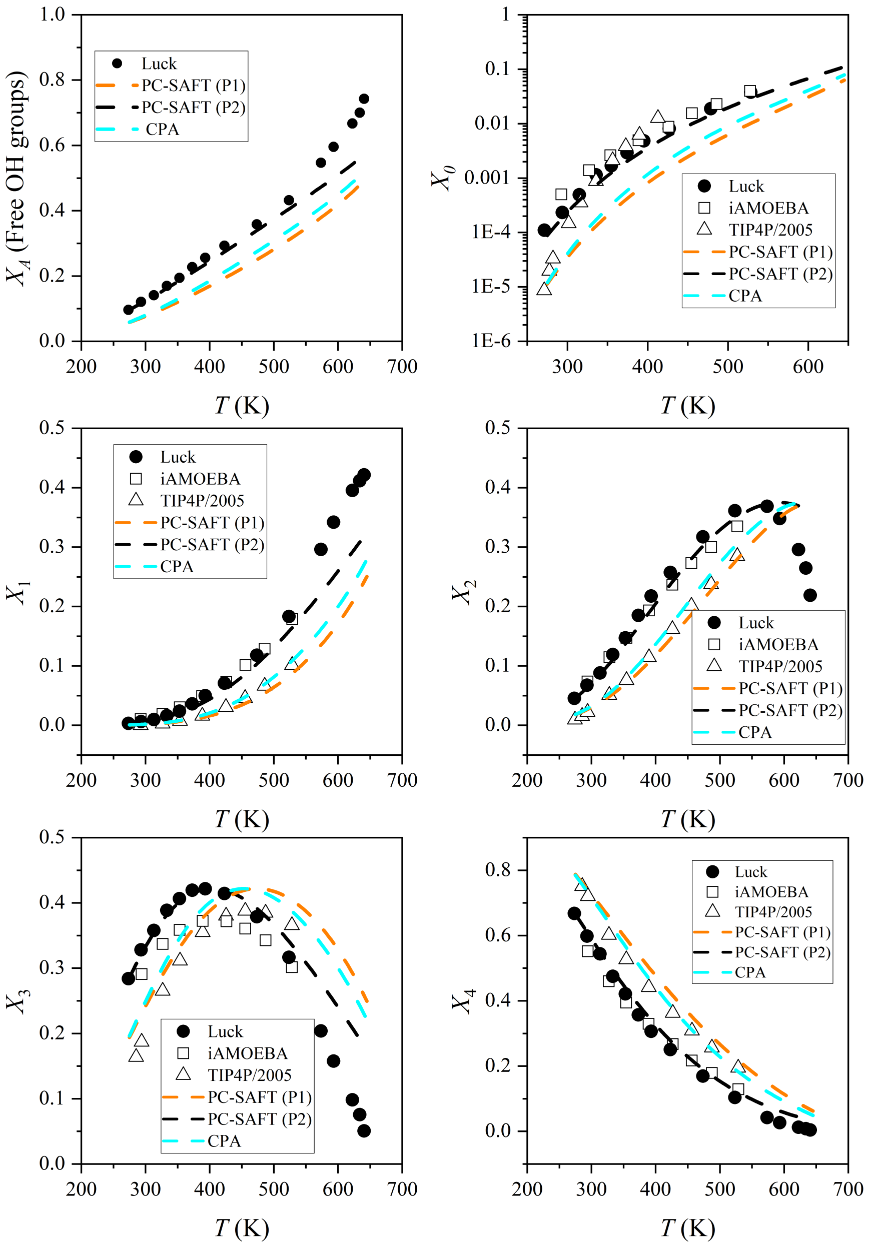

Figure S6: k-times bonded fractions of water at saturated conditions. Symbols are data [8, 9] and dashed lines are results from thermodynamic models.

S5 Supplementary tables

Table S1: Parameter sets of PC-SAFT. In all cases, water is considered to form up to 4 hydrogen bonds.

Set	m	$\sigma(\AA)$	ε/k_B (K)	$\varepsilon^{A_iB_i}/k_B$ (K)	$\kappa^{A_iB_i}$	Reference	Parameter estimation	T range (K)
P1	2.0	2.3449	171.67	1704.06	0.1596	Liang et al. [17]	P^S, ρ^S, fixed $\varepsilon^{A_iB_i}$, fixed $\kappa^{A_iB_i}$, phase equillibria of water-hydrocarbons	280-620
P2	1.0	3.0847	339.5	1538.3	0.003147	Haghmoradi et al. [13]	P^S, ρ^S, fixed m	273-528
P3	2.1945	2.2290	141.66	1804.17	0.2039	Diamantonis and Economou [18]	P^S, ρ^S	275-640

Note: P^S, ρ^S is saturated pressure and saturated liquid density respectively. m is the chain length, σ the temperature independent segment diameter, ε the segment energy parameter, $\varepsilon^{A_iB_i}$ is association energy and $\kappa^{A_iB_i}$ is the association volume.

Table S2: Parameter set of CPA. In all cases, water is considered to form up to 4 hydrogen bonds.

Set	b (L/mol)	Γ (K)	c_1	$\varepsilon^{A_iB_i}/k_B$ (K)	$\beta^{A_iB_i}$ (10^3)	Reference	Parameter estimation	T range (K)
CPA	0.0145	1018.3336	0.6736	2003.1361	69.2	Kontogeorgis et al. [14]	P^S, ρ^S phase equillibria of water-hydrocarbons	278-582

Note: P^S, ρ^S is saturated pressure and saturated liquid density respectively. b is the co-volume parameter, Γ and c_1 are constant parameters related to the temperature dependent $a(T)$, $\varepsilon^{A_iB_i}$ is association energy and $\beta^{A_iB_i}$ is the association volume parameter.

Table S3: Parameter set of SAFT-VR Mie. In all cases, water is considered to form up to 4 hydrogen bonds.

Set	m	σ (Å)	λ_r	λ_a	ε/k_B (K)	$\varepsilon^{A_iB_i}/k_B$ (K)	$K^{A_iB_i}$	Reference	Parameter estimation	T range (K)
SAFT-VR Mie	1.0	3.0063	17.02	6.0	266.68	1985.4	101.69	Dufal et al. [20]	P^S, ρ^S, fixed m	273-615

Note: P^S, ρ^S is saturated pressure and saturated liquid density respectively. m is the chain length, σ the temperature independent segment diameter, λ_r, λ_a are the exponents characterising the repulsive and attractive dispersion interactions respectively, ε the segment energy parameter, $\varepsilon^{A_iB_i}$ is association energy and $K^{A_iB_i}$ is the association volume.

Table S4: LDL fractions from spectroscopy data

Conditions	LDL (tetrahedral) fraction	Technique and author
298K 0.1 MPa	20% ± 20%	X-ray absorption Wernet et al. [12]
363K, 0.1 MPa	15% ± 15%	X-ray absorption Wernet et al. [12]
280K, 0.1 MPa	34.5%	X-ray emission, Tokushima et al. [11]
297K,0.1 MPa	33.3%	X-ray emission, Tokushima et al. [11]
335, 0.1 MPa	27.7%	X-ray emission, Tokushima et al. [11]

a) For Tokushima et al. [11] we have calculated these fractions based on their ratios of distorted/tetrahedral at these conditions b) It should be mentioned that there are also other data that have much lower uncertainty estimates such as the data from Mallamace et al. [6] and Nilsson [7].

Table S5: Fitted k_{ij} values for each parameter set of PC-SAFT. The parameter sets are briefly described in Table S1

Set	Reference	k_{ij}
P1 (PC-SAFT)	Liang et al. [17]	0.0021
P2 (PC-SAFT)	Haghmoradi et al. [13]	0.2095
P3 (PC-SAFT)	Diamantonis and Economou [18]	0.0088

Table S6: Percentage absolute average deviation (% AAD) values for the different fractions and parameters sets, when compared against TIP4P/2005 values from Fouad et al. [9] (saturated conditions). Temperature range: 272-528K. Lowest % AAD values of each column are underlined.

Set	% AAD				
	X_0	X_1	X_2	X_3	X_4
P1 (PC-SAFT)	26.1	277	19.3	12.4	10.3
P2 (PC-SAFT)	313	1235	123	30.2	23.5
P3 (PC-SAFT)	55.1	180	19.4	6.3	20.4
CPA	10.0	300	22.2	14.1	3.9

Table S7: Percentage absolute average deviation (% AAD) values for the different fractions and parameters sets, when compared against iAMOEBA values from Fouad et al. [9] (saturated conditions). Temperature range: 292-528K. Lowest % AAD values of each column are underlined.

Set	% AAD				
	X_0	X_1	X_2	X_3	X_4
P1 (PC-SAFT)	85.7	70.0	40.8	15.8	53.6
P2 (PC-SAFT)	43.3	23.1	5.5	11.7	6.5
P3 (PC-SAFT)	90.6	77.7	50.2	19.3	68.6
CPA	79.8	62.2	33.5	14.5	40.5

Table S8: Percentage absolute average deviation (%AAD) values for the different fractions and parameters sets, when compared against data from Luck [8] (saturated conditions). Temperature range: 273-640 K. Lowest %AAD values of each column are underlined

Set	% AAD
	X_A
P1 (PC-SAFT)	34.7
P2 (PC-SAFT)	9.4
P3 (PC-SAFT)	40.9
CPA	30.1

SI References

[1] Harshad Pathak, JC Palmer, Daniel Schlesinger, Kjartan Thor Wikfeldt, Jonas A Sellberg, Lars GM Pettersson, and Anders Nilsson. The structural validity of various thermodynamical models of supercooled water. *The Journal of chemical physics*, 145(13):134507, 2016.

[2] John Russo and Hajime Tanaka. Understanding water's anomalies with locally favoured structures. *Nature communications*, 5(1):1–11, 2014.

[3] Aswin V Muthachikavil, Baoliang Peng, Georgios M Kontogeorgis, and Xiaodong Liang. Distinguishing weak and strong hydrogen bonds in liquid water—a potential of mean force-based approach. *The Journal of Physical Chemistry B*, 125(26):7187–7198, 2021.

[4] Emily B Moore and Valeria Molinero. Growing correlation length in supercooled water. *The Journal of chemical physics*, 130(24):244505, 2009.

[5] Vincent Holten, Jan V Sengers, and Mikhail A Anisimov. Equation of state for supercooled water at pressures up to 400 mpa. *Journal of Physical and Chemical Reference Data*, 43(4):043101, 2014.

[6] Francesco Mallamace, Caterina Branca, Matteo Broccio, Carmelo Corsaro, Chung-Yuan Mou, and Sow-Hsin Chen. The anomalous behavior of the density of water in the range 30 k¡ t¡ 373 k. *Proceedings of the National Academy of Sciences*, 104(47):18387–18391, 2007.

[7] A. Nilsson. Origin of the anomalous properties in supercooled water. *J. Non-Cryst. Solids*, 2022.

[8] Werner AP Luck. A model of hydrogen-bonded liquids. *Angewandte Chemie International Edition in English*, 19(1):28–41, 1980.

[9] Wael A Fouad, Le Wang, Amin Haghmoradi, D Asthagiri, and Walter G Chapman. Understanding the thermodynamics of hydrogen bonding in alcohol-containing mixtures: cross-association. *The Journal of Physical Chemistry B*, 120(13):3388–3402, 2016.

[10] Aswin V Muthachikavil, Qun Lei, Baoliang Peng, Georgios M Kontogeorgis, and Xiaodong Liang. Structural characteristics of low density environments in liquid water. *Phys. Rev. E*, 105(3):034604, 2022.

[11] Takashi Tokushima, Yoshihisa Harada, Osamu Takahashi, Yasunori Senba, Haruhiko Ohashi, Lars GM Pettersson, Anders Nilsson, and Shik Shin. High resolution x-ray emission spectroscopy of liquid water: The observation of two structural motifs. *Chemical Physics Letters*, 460(4-6):387–400, 2008.

[12] Ph Wernet, D Nordlund, Uwe Bergmann, M Cavalleri, M Odelius, H Ogasawara, L A Naslund, TK Hirsch, L Ojamae, P Glatzel, et al. The structure of the first coordination shell in liquid water. *Science*, 304(5673):995–999, 2004.

[13] Amin Haghmoradi, Deepti Ballal, Wael A Fouad, Le Wang, and Walter G Chapman. Combination of monovalent and divalent sites on an associating species: Application to water. *AIChE Journal*, 67(3):e17146, 2021.

[14] Georgios M Kontogeorgis, Iakovos V Yakoumis, Henk Meijer, Eric Hendriks, and Tony Moorwood. Multicomponent phase equilibrium calculations for water–methanol–alkane mixtures. *Fluid Phase Equilibria*, 158:201–209, 1999.

[15] JL Heidman, C Tsonopoulos, CJ Brady, and GM Wilson. High-temperature mutual solubilities of hydrocarbons and water. part ii: Ethylbenzene, ethylcyclohexane, and n-octane. *AIChE journal*, 31(3):376–384, 1985.

[16] Jiri Polak and Benjamin C-Y Lu. Mutual solubilities of hydrocarbons and water at 0 and 25 c. *Canadian Journal of Chemistry*, 51(24):4018–4023, 1973.

[17] Xiaodong Liang, Ioannis Tsivintzelis, and Georgios M Kontogeorgis. Modeling water containing systems with the simplified PC-SAFT and CPA equations of state. *Industrial & Engineering Chemistry Research*, 53(37):14493–14507, 2014.

[18] Nikolaos I Diamantonis and Ioannis G Economou. Evaluation of statistical associating fluid theory (saft) and perturbed chain-saft equations of state for the calculation of thermodynamic derivative properties of fluids related to carbon capture and sequestration. *Energy & Fuels*, 25(7):3334–3343, 2011.

[19] Frédéric Caupin and Mikhail A. Anisimov. Thermodynamics of supercooled and stretched water: Unifying two-structure description and liquid-vapor spinodal. *Journal of Chemical Physics*, 151(3), 2019.

[20] Simon Dufal, Thomas Lafitte, Andrew J Haslam, Amparo Galindo, Gary NI Clark, Carlos Vega, and George Jackson. The A in SAFT: developing the contribution of association to the helmholtz free energy within a wertheim tpt1 treatment of generic mie fluids. *Molecular Physics*, 113(9-10):948–984, 2015.
